# Targeted DNA Sequencing for Tailored Therapies in Children with Extracranial Solid Tumors

**DOI:** 10.3390/ijms262311463

**Published:** 2025-11-26

**Authors:** Nataliya A. Andreeva, Tatyana V. Shamanskaya, Denis Y. Kachanov, Nikolay V. Zhukov, Nina V. Gegeliya, Ruslan H. Abasov, Natalia Y. Usman, Anastasia V. Protsvetkina, Dmitry M. Konovalov, Dmitry V. Litvinov, Natalia V. Myakova, Nikolay S. Grachev, Galina A. Novichkova, Alexander E. Druy

**Affiliations:** 1 Dmitry Rogachev National Medical Research Center of Pediatric Hematology, Oncology and Immunology, Moscow 117198, Russia; shamanskaya.tatyana@gmail.com (T.V.S.); kachanov78@gmail.com (D.Y.K.); 1cancerdoctor1@gmail.com (N.V.Z.); gegeliya.nina@dgoi.ru (N.V.G.); ruslan.abasov.2013@mail.ru (R.H.A.); natalia.usman@yandex.ru (N.Y.U.); a.procvetkina@dgoi.ru (A.V.P.); dmk_nadf@mail.ru (D.M.K.); litvinov_d_v@mail.ru (D.V.L.); nmiakova@mail.ru (N.V.M.); nick-grachev@yandex.ru (N.S.G.); gnovichkova@yandex.ru (G.A.N.); dr-drui@yandex.ru (A.E.D.); 2 Research Institute of Medical Cell Technologies, Yekaterinburg 620026, Russia

**Keywords:** precision treatment, targeted treatment, molecular therapy, NGS, DNA sequencing, children, neuroblastoma

## Abstract

Next-generation sequencing (NGS) is instrumental for clinical decisions on molecularly targeted therapy (TT). In pediatric oncology, TT is a relatively rare choice administered chiefly on a tumor-agnostic basis. The investigation enrolled 304 pediatric patients with extracranial solid tumors that were diagnosed and treated in 2018–2023. Tumor DNA was sequenced using a customized QiaSeq panel (Qiagen, Hilden, Germany) of genes known to be relevant for pediatric solid tumors, including *ALK*, *BRAF*, *BRCA1/2*, *EGFR*, *FGFR1*, *KIT*, *MAP2K1/2*, *NF1*, *PDGFRA/B*, *PIK3CA*, *PTEN*, *PTPN11*, RAS family genes, etc. The assay allowed detection of nucleotide substitutions and small insertions/deletions, as well as gene copy number alterations. TT sensitivity predictors were identified in 120/304 cases (39.5%): Tier II in 83 patients, Tier IB in 32 patients (almost always ALK in neuroblastoma, *n* = 31) and Tier IA in 5 patients: BRAF p.V600E (*n* = 3) and NF1 aberrations (*n* = 2). TT commenced in 21/304 cases (6.9%), often first-line or as a first relapse therapy (14/21 cases), combined with chemotherapy (TT-CT) in 13 cases. The median of TT duration was 10.9 (range 0.8–43.5) months for single-mode and 12.3 (0.3–61.5) months for TT-CT. Clinical benefit rate was achieved in 14/21 patients (66.7%). At the time of writing, nine patients (42.8%) have no progression and are still on treatment for 30.4 months (range 10.3–40.5) after the start of TT. The median time to the best response to TT was 6 (range 0.8–12.3) months. The tolerance was generally good: the therapy was discontinued for toxicity in only one case. The study provides a TT-focused prospective analysis still rare in pediatric oncology. The outcomes indicate satisfactory tolerance and promising efficacy of TT, prompting an update of current treatment standards for several pediatric cancers.

## 1. Introduction

Extracranial solid tumors of childhood are rare conditions of varying malignancy, heterogeneous in terms of histogenesis, molecular pathogenesis, clinical course and prognosis. Unfortunately, till now, the prognosis for metastatic and relapsed/refractory pediatric solid cancers treated by conventional cytotoxic therapy remains unfavorable. The most common extracranial solid tumors in children are neuroblastoma and soft-tissue and bone sarcomas. Neuroblastoma exhibits extremely heterogeneous clinical behavior and has variable prognosis. The worst prognosis is seen in patients over 18 months of age with metastatic stage four or stage M disease, especially in the presence of *MYCN* gene amplification. Various therapeutic modalities are used for high-risk neuroblastoma, including multicomponent chemotherapy, high-dose chemotherapy with autologous hematopoietic stem cell rescue, immunotherapy, radiotherapy, and surgery. Despite the highly intensive therapies, the 5-year event-free survival rate does not exceed 50–60% [1]. Patients with soft-tissue sarcomas also receive intensive multicomponent chemotherapy, radiotherapy, surgery, and at advanced stages, also have a dismal outcome, with survival rates below 30% [2]. It should be noted that the multimodal treatment of the primary tumor significantly limits the therapeutic options in case of disease relapse or progression. To date, there are no established protocols of second-line treatment for the vast majority of pediatric cancers. This indicates the importance of the search for alternative treatment modalities, including targeted therapies (TTs), which require mandatory identification of the molecular target.

The majority of pediatric solid tumors directed for advanced molecular genetic investigation are tumors with exhausted standard treatment options, including high-risk neuroblastoma (NB), metastatic soft-tissue sarcomas and miscellaneous rare tumors with ≤40–50% long-term survival after first-line standard therapy and no efficacious second-line modalities [1,2,3,4,5,6]. Molecularly targeted therapy provides a complementary clinical strategy in such patients. Somatic mutation burden in pediatric tumors is generally low; however, up to 30–45% of the cases harbor molecular markers of TT sensitivity [7,8,9,10]. In pediatric oncology, the incidence of common actionable markers for a particular entity is too low to ensure the development of specific indications for TT; accordingly, TT remains an experiential choice, most often as a salvage therapy.

The implementation of TT in children is based on the experience and reported evidence of its efficacy in adult cancers. However, due to specific biological features of childhood tumors, the efficacy may vary. Currently, the evidence of TT benefits in pediatric tumors is extremely limited, particularly in connection with the limited enrollment opportunities.

Kinase gene rearrangements represent valid targets for TT; however, their incidence is limited and restricted to particular tumor types [8]. The vast majority of pediatric cancers harbor single-nucleotide pathogenic mutations, the predictive significance of which remains poorly investigated.

This investigation aimed at evaluating the clinical significance for genetic variants identified by targeted next-generation sequencing (NGS) of tumor DNA in pediatric solid tumors, and primary validation of these variants as molecular markers of sensitivity to molecularly targeted therapy.

## 2. Results

### 2.1. Enrollment Patients

The study enrolled 304 patients with median age 4.2 (range 0–17.6) years at diagnosis (Table 1). Patients with neuroblastoma (NB, 55.3%) or rhabdomyosarcoma (RMS, 15.8%) were routed to NGS both in recurrent/refractory cases and in the presence of high-risk disease. Tumor material from patients with other entities (non-RMS soft-tissue sarcoma, nephroblastoma, Ewing sarcoma) was routed for molecular genetic study, predominantly at recurrence. The group of non-RMS soft-tissue sarcomas contained three cases of spindle-cell neoplasms harboring no fusion transcripts as defined by targeted RNA sequencing (TrueSight RNA Fusion panel, Illumina, San Diego, CA, USA).

Unresectable tumors that were non-responsive to preoperative chemotherapy provided the key rationale for NGS in 11.2% of the cohort; these were mostly soft-tissue sarcoma (STS) or carcinoma cases where adequate local control was clinically crucial and a neoadjuvant TT could promote regression of the tumor sufficient to render it resectable.

The most common were genetic markers of potential clinical significance assigned to Tier IIC, including actionable markers (revealed in 15.6% of cases), whereas Tier IA–B markers events were relatively rare (Table 2).

### 2.2. TT Sensitivity Markers

Tier IA predictors were identified in two cases of melanoma and low-grade spindle-cell sarcoma (*BRAF* p.V600E, three patients) and plexiform neurofibroma (*NF1* aberrations, two patients). Tier IB predictors were identified in NB (*ALK* aberrations, 31 patients) and gastrointestinal stromal tumor (GIST, KIT amplification, one patient). Tier IIC–D predictors were non-specific and found in various tumors, including NB, STS, malignant rhabdoid tumor, nephroblastoma, carcinomas, Ewing sarcoma, osteosarcoma, GIST and benign neoplasms (83 patients). The variants included activating missense mutations in protein kinase-encoding genes *ALK*, *PDGFRA/B*, *HRAS*, *PIK3CA*, *MAP2K1*, *FGFR1*, *EGFR*, *RET*, *KIT*, *MET* and loss-of-function events affecting DNA repair system genes *BRCA1/2* and *MLH1*.

Genetic variants predicting TT efficacy at various tiers of clinical significance were identified in 120 patients, ultimately affording TT prescription in 21 cases (17.5%). Predictors assigned with high clinical significance (Tier I, 37 patients) warranted TT in 17 cases (45.9%).

In view of the non-standardized status of TT in pediatric settings, before the therapy initiation, a comprehensive discussion was held with parents or legal representatives of the patient concerning the potential benefits and risks of the treatment. Additional informed consent was obtained for the experimental treatment in each particular case of the study.

*BRAF* p.V600E warranted a combination therapy by dabrafenib–trametinib in patients with melanoma and with low-grade spindle-cell STS. In both cases, BRAF and MEK inhibition resulted in deep tumor response; however, disease progression was noted 15 and 7 months, respectively, from TT initiation. Another Tier IA predictor, tumor-specific NF1 aberration, warranted TT with selumetinib in a patient with unresectable plexiform neurofibroma, resulting in disease stabilization.

Within the framework of this study, TT was most typically prescribed on the basis of Tier IB predictors (*n* = 13), predominantly *ALK* aberrations in NB, including missense mutations in 10 patients and gene amplification in 3 patients. (These results will be discussed separately.) TT with imatinib was administered in a single case of GIST, *KIT*-amplified, which allowed short-term stabilization of the tumor. A subsequent local progression of the disease was totally resected and multikinase-inhibitor sunitinib was administered as a maintenance therapy.

Lower-rank predictors of potential clinical significance (Tier II) were implemented as an indication for TT in isolated cases. In two patients with myoepithelial carcinoma, *RET* p.H926L and *FGFR1* amplification warranted vandetanib and lenvatinib, respectively. Specifically, the vandetanib therapy halted an actively progressing myoepithelial carcinoma with *RET* p.H926L, affording 35 months of continuous disease stabilization. The therapy was discontinued without signs of disease progression. In contrast, TT of *FGFR1*-amplified myoepithelial carcinoma with lenvatinib was unsuccessful. Similarly, *HRAS* p.Q61L in ectomesenchymoma was clinically accepted as a marker of sensitivity to MEK-inhibitor trametinib, but this therapy failed to provide a clinical benefit.

**Figure 1:**
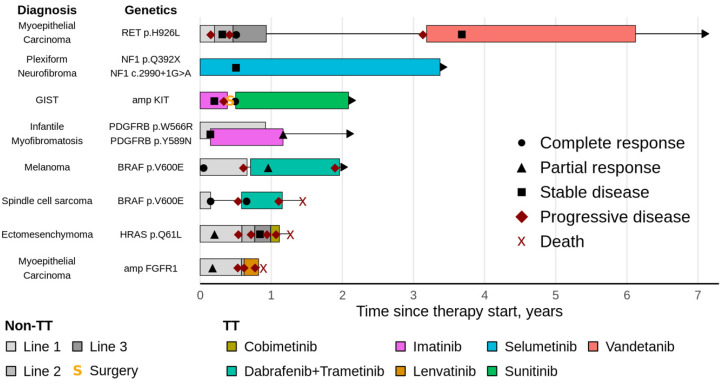
Clinical implementation of TT sensitivity predictors with regard to diagnosis, drug choice, treatment schedules and clinical outcomes for the cohort except neuroblastoma cases. TT—targeted therapy, non-TT—without targeted therapy, GIST—gastrointestinal stromal tumor, amp—amplification.

The combination of *PDGFRB* p.W566R/p.Y589N variants warranted imatinib for advanced infantile myofibromatosis with the liver, intraventricular septum involvement and multiple bone lesions. Chemotherapy with vinblastine and methotrexate for 9 weeks only allowed disease stabilization and was complicated by acute heart failure. Subsequent complementation of chemotherapy with imatinib resulted in significant clinical improvement and objective tumor response. After 9 months of combined therapy, TT was continued for 1 year. The summary of clinical parameters of TT recipients is presented in Table 3; the median age of diagnosis in this group was 3.7 years (range 0.1–14.8). A graphic summary of TT recipients with non-neuroblastoma conditions is given in Figure 1. As patients with neuroblastoma prevailed among TT recipients, they were discussed separately.

### 2.3. TT in Neuroblastoma

Patients with NB (*n* = 168) were routed to NGS as the means of search for TT sensitivity predictors, either upon stratification to high-risk group at primary diagnosis (72 patients) or in the event of recurrence/progression/refractory course (*n* = 96). TT sensitivity predictors according to the AMP/ASCO/CAP Guidelines were identified in 66/168 cases (39.3%), including Tier IB markers in 31 patients (18.5%, all involving *ALK*: activating missense variants in 21 patients and gene amplifications in 10 patients); Tier IIC in 21 patients (12.5%, affecting *BRCA1/2*, *BRAF*, *PDGFRA/B*, *TSC1*, *POLD1*), Tier IID in 14 patients (8.3%, mostly *MET*, *KIT*, *MAP2K1*, *EGFR*, *RET*, *BRAF*, *PDGFRB* amplifications). Molecularly targeted intensification of standard treatment was administered in 13 cases harboring ALK aberrations. The scope of ALK inhibitors included crizotinib (four patients), alectinib (one patient), ceritinib (four patients) and lorlatinib (five patients). The treatment continued 13.2 (0.3–60.9) months.

Four patients with high-risk NB received ALK inhibitors first-line in combination with standard therapy: either in parallel with induction chemotherapy (two patients) or at consolidation phase after high-dose chemotherapy (two patients). Upon completion of the standard protocol, single-mode TT was continued. By the time of writing, the therapeutic effect was sustained in 4/4 cases, amounting to very good partial response (VGPR) in two patients and partial response (PR) in two patients. TT has been administered for 10.3–39.9 months (median 25.1 months) and continued in single mode in three out of four recipients (in one patient, lorlatinib was discontinued from 10.3 months since the commencement due to gastrointestinal toxicity grade 4).

Nine patients with NB received ALK inhibitors for the first or subsequent recurrence/progression. In one case, crizotinib commenced in a patient with *ALK* p.R1275Q, developing the disease at an age of 9.2 years, as a maintenance therapy during partial response to anti-relapse cycles of irinotecan and temozolomide. The disease stabilized for 43.5 months on crizotinib, after which a metastatic progression was recorded. In eight cases, ALK inhibitors commenced as a component of various anti-recurrence schemes of chemotherapy; the treatment was efficacious in two patients (25%), affording disease stabilization and PR lasting 26.6 and 61.5 months, respectively.

**Figure 2:**
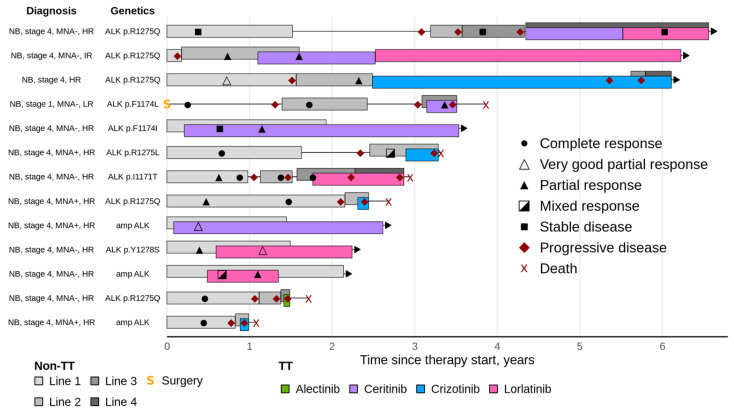
Clinical implementation of TT sensitivity predictors in neuroblastoma with regard to diagnosis, drug choice, treatment schedules and clinical outcomes. TT—targeted therapy, non-TT—without targeted therapy, NB—neuroblastoma, HR—high-risk group, IR—intermediate-risk group, LR—low-risk group, MNA—MYCN amplification, amp—amplification.

A graphic summary of TT for NB is given in Figure 2. Six patients with NB continue to receive TT with sustained clinical response at the time of this writing.

### 2.4. Outcomes in TT Recipients

Overall, TT was administered first-line, or at first treatment failure, in over half of the cases (14/21, 66.7%). To potentiate the effect of standard protocols, molecularly targeted drugs were often combined with chemotherapy (13/21, 61.9%). TT as monotherapy (8/21 cases, 38.1%) was used mostly at recurrence or progression as a salvage therapy based on TT sensitivity predictors of high (Tier I, five patients) or potential (Tier II, three patients) clinical significance. The mono and combined TT regimens continued for median 10.9 (range 0.8–43.5) and 12.3 (range 0.3–61.5) months, respectively, to afford partial objective response rates of 37.5% (3/8 cases) and 53.8% (7/13 cases), respectively. The overall objective response to TT was 47.6% (10/21 cases). Prolonged stabilization of the disease (over 6 months) was achieved in 4/21 cases (19.0%), mostly on monotherapy (3/4 cases). The median time to best response (including disease stabilization on TT) was 6 months (range 0.8–12.3 months). At the time of writing, the therapeutic effect persists in 9/21 cases (42.8%; 30.4 (10.3–40.5) months on TT), with objective response sustained in 6/21 cases (28.6%; 25.1 (10.3–61.5) months on TT) and stabilization in 3/21 cases (14.3%; 35.3 (26.6–40.5) months on TT). Disease progression on TT occurred in 12/21 cases (57.2%) including seven non-responders, four after best response and one after stabilization. Remarkably, the association of progression events with the tier of predictor that warranted TT administration appeared weak, with higher progression incidence rates for Tier IB predictors in NB and GIST.

### 2.5. TT-Related Adverse Events

Toxicity complications of grade 3–4 associated with TT were encountered in 6/21 cases (28.6%).

The dabrafenib–trametinib combination administered in two patients with *BRAF* p.V600E tumors entailed adverse events of grade 3–4, including skin rashes, pyrexia and a hydrothorax episode (in a patient with pelvic spindle-cell STS), necessitating dose reduction and therapy breaks in both cases. Patients with NB on a prolonged crizotinib monotherapy developed a generalized edema of grade 3 at 27 months since the commencement, necessitating a reduction in the dose by 30%. In one case, ceritinib monotherapy was complicated by hepatotoxicity of grade 3 (elevated liver enzymes at 17 months since the commencement); this was resolved by switching to lorlatinib. Patients with chronic colitis and short bowel syndrome developed gastrointestinal toxicity of grade 4 on lorlatinib, necessitating breaks in the regimen and the eventual discontinuation of TT 10 months later. Another patient developed hypercholesterolemia of grade 3 after 10 months on lorlatinib, resolved by dose reduction.

## 3. Discussion

Molecular genetic investigations, notably by DNA/RNA sequencing methods, are increasingly applied in clinical practice; the objectives include diagnosis specification, search for actionable markers and tumor biology insights. Targeted DNA sequencing reveals nucleotide substitutions and small indels with high accuracy and can suggest copy number variations for particular regions; hence, its priority as the method of choice for clinical use in oncology. Targeted DNA sequencing is ‘blind’ to certain structural variants epitomized by chimeric gene expression units; such cases require identification of fusion transcripts by RNA sequencing [11,12]. Still, the majority of molecular predictors for TT (except the actionable protein kinase gene rearrangements) are nucleotide variants which are perfectly detectable by tumor DNA sequencing.

Enrollment in the current study was aimed at searching for druggable targets in high-risk tumors with anticipated low efficacy of standard therapy and/or progression/recurrence/refractory course of the disease. The current pediatric use of TT is largely confined to few agnostic standards including nivolumab and pembrolizumab for tumors with high mutation burden; dabrafenib/trametinib combinations for *BRAF* p.V600E-mutated tumors; and entrectinib and larotrectinib for NTRK-rearranged tumors [13]. In this regard, accumulation of knowledge on the use of TT in pediatric cohorts based on specific mutational profiles is of particular value. To date, several research teams have published the results of DNA and RNA profiling of pediatric tumors (iTHER, INFORM, ZERO, MAPPYACTS, Ped-MATCH, KiCS), reporting a 32–67% incidence of actionable alterations defined in accordance with local criteria, ensuing TT administration in 13–29% of the patients [14,15,16,17,18,19]. In this report, we applied a targeted sequencing panel to tumor DNA to reveal actionable variants in 120/304 cases (39.5%). Accordingly, TT was administered in 21/304 cases (6.9%), i.e., at substantively lower rates compared with other studies, which can be attributed to the enhanced local criteria for TT, namely the requirement of published evidence on the efficacy of particular drugs in children or the exhaustion of all standard curative options.

The incidence of fully validated actionable markers in pediatric solid tumors is generally low, and the presence of targetable mutations is not typical for childhood tumors [8]. However, in some tumor types, the incidence of targetable genetic aberrations is much higher. Particular types of pediatric malignancies (infantile fibrosarcoma, NTRK-rearranged spindle-cell neoplasm, infant-type hemispheric glioma) harbor gene fusion-based druggable alterations in nearly all cases. The actionable point mutations are more frequently presented in neuroblastoma (*ALK* missense mutations) and prevail in high-risk and relapse tumors, warranting TT with ALK inhibitors [20,21]. Nevertheless, the majority of molecular alterations in pediatric cancers, including *MYCN* amplification and segmental chromosomal aberrations in neuroblastoma, and *TP53*, *MYOD1* and *RAS* family gene mutations in soft-tissue sarcomas, provide no rationale for TT with available medications [1,22].

In the studied cohort, Tier IA predictors were rare (agnostic *BRAF* p.V600E and tumor-specific *NF1* aberrations), while Tier IB–II predictors (i.e., mentioned in single studies on possible benefits of TT and/or warranting enrollment in a clinical trial for TT in other tumor types, mostly adult) were common, including *ALK*, *BRCA1/2*, *PIK3CA*, *PDGFRB*, *TSC2* and *KIT* variants.

Of extracranial pediatric solid tumors, actionable markers are most commonly found in NB. *ALK* variants that were clinically targetable with ALK inhibitors were identified by us in 18.5% of high-risk NB. Other activating events (e.g., in *BRAF*, *PDGFRA/B*, found in solitary NB samples) were dismissed and TT was implemented for ALK-aberrated cases only. A first-generation drug, crizotinib, was applied chiefly in patients with *ALK* p.1275^mut^ to limited efficacy (disease stabilization achieved in 1/4 patient), consistent with published data. Foster et al. (2021) reported a 15% partial objective response to crizotinib in recurrent/refractory NB (3/20 cases) [20]. More advanced inhibitors (ceritinib, lorlatinib) covering a broader spectrum of *ALK* variants were administered in 9/13 cases of TT for NB (69.2%). Combining these drugs with chemotherapy and/or immunotherapy with dinutuximab beta afforded an objective response of 66.7% (6/9 cases), albeit to stabilization rates of ~10% only (1/9 patient). A similar objective response of 62% was reported by Goldsmith et al. for lorlatinib in combination with chemotherapy (temozolomide/topotecan) [21]. It should be noted that the prevailing use of TT as a component in combination regimens impedes the objective evaluation of efficacy for ALK inhibitors per se. At the same time, considering the tolerable toxicity and good efficacy of the combination regimens, the reinforcement of conventional options with TT, particularly first-line options, is certainly a promising strategy. The efficacy of ALK inhibitors in primary patients with high-risk NB-harboring *ALK* aberrations is currently a subject of prospective clinical trials (NCT03126916).

Although TT was generally well-tolerated in our setting, some patients experienced severe adverse events requiring dose adjustment or “drug holidays”. In most cases, we encountered typical side effects of each particular targeted medication, known from adult oncology practice. For dabrafenib and trametinib therapy, the most common side effects reported in both adults and children (CTMT212X2101) include pyrexia, fatigue, rashes, chills, headache, hemorrhage, cough, edema, etc. [23]; most of these effects were observed in the current study.

ALK inhibitor therapy has a variable toxicity spectrum depending on the drug generation. While using crizotinib, our patient developed generalized edema, which is a common side effect of crizotinib therapy in adults [24]. Crizotinib and ceritinib have been associated with hepatotoxicity [25] in contrast with lorlatinib, which warranted a switch from ceritinib to lorlatinib in one patient with neuroblastoma. However, lorlatinib demonstrates a specific profile of adverse events, including hypercholesterolemia and hypertriglyceridemia, observed in both adults and in children [21,26]. The most serious side effect of lorlatinib in adults is central nervous system toxicity, including impaired cognitive function and other mental health problems. It is difficult to assess this type of adverse event in young children.

Categorization of predictive biomarkers for TT is largely situational, reflecting the current volume of clinical data on therapy for particular aberration(s). For extremely rare conditions, e.g., infantile myofibromatosis, TT experience comprises isolated clinical observations. *PDGFRB* variants of Tier IIC identified in a substrate of infantile myofibromatosis provided indications for imatinib, which afforded significant reduction in pathological foci and the elimination of a life-threatening lesion in the interventricular septum. RAS genes are most commonly mutated in RMS and NB; however, successful clinical experience of targeting hotspot mutations in RAS for these tumors is missing. In our clinical setting, TT with MEK-inhibitor cobimetinib in a patient with ectomesenchymoma-harboring *HRAS* p.R61Q genetic variant leading to RAS–RAF–MEK pathway activation was unsuccessful.

Typing of the tumors for distinct molecular genetic signatures should be established as a clinical diagnostic standard in pediatric oncology, especially given the ephemeral guidelines for TT in pediatric practice, amid continually updated scopes of available targeted medications. The strong diagnostic capacity of NGS, which is increasingly accurate, affordable and integral to clinical decision-making, should be fully employed in ordinary clinical settings in order to optimize the treatment outcomes in terms of controlled efficacy/toxicity rates, quality of life and survival with pediatric cancers.

## 4. Materials and Methods

The investigation was approved by the local Ethical Committee of the Dmitry Rogachev National Medical Research Center of Pediatric Hematology, Oncology and Immunology. All patients or their legal representatives provided voluntary informed consent for participation in the study.

Targeted NGS using a customized QiaSeq panel (Qiagen, Germany) was applied to DNA isolated from formalin-fixed paraffin-embedded tumor tissues of patients receiving treatment at the Dmitry Rogachev National Medical Research Center of Pediatric Hematology, Oncology and Immunology in 2018–2023. Tumor material was preliminarily assessed by two pathologists and specimens with >60% of tumor cells were analyzed.

Nucleic acids extraction was performed with AllPrep DNA/RNA FFPE Kit (Qiagen) or RNA/DNA Purification Kit (Norgen, Thorold, ON, Canada). The concentration of extracted DNA was assessed with Nanodrop One spectrophotometer (Thermo Fischer Scientific, Waltham, MA, USA) and Qubit fluorometer using Qubit dsDNA BR Assay Kit (Thermo Fischer Scientific). Subsequently, DNA was treated with NEBNext FFPE DNA Repair v2 Module (New England Biolabs, Ipswich, MA, USA).

The targeted panel included coding sequences and canonical splice sites (according to MANE Select transcripts) of genes involved in molecular pathogenesis of pediatric solid tumors as well as harboring actionable mutations (Supplementary Materials). NGS was carried out with MiSeq sequencing kit v.2 300× in a MiSeq instrument (Illumina, USA) using a paired-end mode to a >1000× mean coverage depth of target regions. Genetic variants were considered for interpretation and reporting if the variant allele frequency was above 5%. The clinical relevance of identified genetic variants was assessed using VarSome Premium, COSMIC, OncoKB databases for somatic variants and literature data. The identified diagnostic/prognostic/actionability markers were classified in accordance with the AMP/ASCO/CAP Guideline recommendations [27]. Gene copy number evaluations used Qiagen GeneGlobe hub (QIAgen, Hilden, Germany). Clinically significant copy number anomalies were validated by FISH (Fluorescence In Situ Hybridization) and/or MLPA (Multiplex Ligation-dependent Probe Amplification) methods prior to being used clinically. When available, relapse/progression tumor tissue was used for analysis (*n* = 64) to account for actual mutational landscapes.

Referral of tumor material for NGS was made by the treating physician or the medical council. Indications for the molecular characterization of the tumor included either:

(1) Tumors with known role of nucleotide variants in molecular pathogenesis.
(2) Recurrence/progression of the disease, including situations of exhausted therapeutic options, given a good patient’s somatic status (Karnofsky index >50%) and >6 months life expectancy.
(3) High-/ultra-high-risk disease by stratification according to current protocols.
(4) Inoperability, including benign neoplasms deteriorating the quality of life.

Exclusion criteria were central nervous system tumors, hematological tumors and tumors with known or suspected chimeric molecular drivers liable to RNA sequencing.

NGS was carried out with MiSeq sequencing kit v.2 300× in a MiSeq instrument (Illumina, USA) using a paired-end mode to a >1000× mean coverage depth of target regions. Genetic variants were considered for interpretation and reporting if variant allele frequency was above 5%. The clinical relevance of identified genetic variants was assessed using VarSome Premium, COSMIC, OncoKB databases for somatic variants and literature data. The identified diagnostic/prognostic/actionability markers were classified in accordance with the AMP/ASCO/CAP Guideline recommendations [11]. Gene copy number evaluations used Qiagen GeneGlobe hub (QIAgen, Germany). Clinically significant copy number anomalies were validated by FISH (Fluorescence In Situ Hybridization) and/or MLPA (Multiplex Ligation-dependent Probe Amplification) methods prior to being used clinically. When available, relapse/progression tumor tissue was used for analysis (*n* = 64) to account for actual mutational landscapes.

The identified genetic variants of clinical significance were considered by an interdisciplinary molecular tumor board. Identification of actionable variants in patients experiencing a continued response to standard regimens (NB2004 protocol enforced with dinutuximab beta, CWS2009 protocol, etc.), first-line or anti-recurrence, and entailed no TT prescription, which was reserved for progression/recurrence. However, four patients with high-risk NB received ALK inhibitors in parallel with first-line chemotherapy (CT). Prior to TT, the patients and their parents/guardians were informed on potential advances and risks of the proposed treatment. All patients or their legal representatives provided voluntary informed consent for TT. The dose and schedule of appointed medication was determined according to approved prescribing information or based on the published results of TT in pediatric patients. TT continued until disease progression of unacceptable toxicity.

The response to therapy was evaluated in accordance with international criteria stipulated by treatment protocols for specific tumor types. Medical imaging (computed tomography and/or magnetic resonance imaging) was performed every 3 months (off-schedule if disease progression suspected). The toxicity was evaluated according to the Common Terminology Criteria for Adverse Events (CTCAE, Version 5).

## 5. Conclusions

Study of mutational landscapes in high-risk pediatric solid cancers is a mandatory step when considering a molecularly tailored therapy. DNA sequencing is the primary method of choice for this purpose in the majority pediatric cancers, allowing identification for the various of actionable variants.

Here, we report a ~40% incidence of actionable molecular markers Tier I–II in a diverse pediatric cohort with extracranial solid malignancies, revealed by targeted NGS. With a prerequisite record on pediatric efficacy for a particular drug, TT was administered in 6.3% of the cases, with or without concomitant chemotherapy, to an objective response rate of 42.8%. At median TT duration of 25.1 months (10.3–61.5 months), sustained response was achieved in 28.6% of the recipients. The outcomes are fairly promising in terms of prospective clinical algorithms involving TT as a standard option in pediatric solid cancers.

Considering the rarity and molecular heterogeneity of solid tumors in children and adolescents, further progress with TT in primary and refractory pediatric cancers will require planned cooperation on a multicenter scale, with coordinated enrollment and authorized registries facilitating collection and management of data on the use of targeted drugs in pediatric clinical practice. The long-term outcomes of TT in children remain understudied.

## Figures and Tables

**Table 1:** Structure of the cohort by diagnosis and clinical routing.

Parameters		Total (%)	Objectives for NGS *		
			High-Risk Group	Recurrent/Refractory Course	Unresectable Tumor #
Quantity		304 (100.0%)	134 (44.1%)	136 (44.7%)	34 (11.2%)
Diagnosis	NB *	168 (55.3%)	72 (42.9%)	96 (57.1%)	0
	RMS *	48 (15.8%)	33 (68.7%)	13 (27.1%)	2 (4.2%)
	soft-tissue tumors, non-RMS	29 (9.5%)	9 (31.0%)	6 (20.7%)	14 (48.3%)
	nephroblastoma	13 (4.3%)	5 (38.5%)	8 (61.5%)	0
	carcinomas	13 (4.3%)	6 (46.1%)	3 (23.1%)	4 (30.8%)
	Ewing sarcoma	6 (2.0%)	2 (33.3%)	4 (66.7%)	0
	other malignancies ^†^	20 (6.5%)	7 (35.0%)	7 (35.0%)	6 (30.0%)
	benign tumors ^‡^	7 (2.3%)	0	0	7 (100%)

# Unresectable tumors outside high-risk groups as defined by conventional stratifications schemes. * NGS—next-generation sequencing, NB—neuroblastoma, RMS—rhabdomyosarcoma; † germ-cell tumor (*n* = 3), hepatoblastoma (*n* = 2), melanoma/nevus (*n* = 4), neuroendocrine tumor (*n* = 1), gastrointestinal stromal tumor (*n* = 3), osteosarcoma (*n* = 3), pleuropulmonary blastoma (*n* = 3), malignant giant-cell tumor (*n* = 1). ‡ plexiform neurofibroma (*n* = 2), infantile myofibroma (*n* = 2), pneumocytoma (*n* = 1), lymphangioendothelioma (*n* = 1), ectomesenchymal chondromyxoid tumor (*n* = 1).

**Table 2:** Clinically significant variants identified by targeted NGS, structured by clinical implication and significance with regard to histological diagnosis.

Diagnostic Entities		*n*	TT *-Sensitivity Predictors			
			IA	IB	IIC	IID
Total		304 (100.0%)	5 (1.5%)	32 (9.6%)	52 (15.6%)	31 (9.3%)
Diagnosis	NB *	168 (50.3%)	0	31 (18.5%)	21 (12.5%)	14 (8.3%)
	RMS *	54 (16.2%)	0	0	11 (20.4%)	10 (18.5%)
	soft-tissue tumors, non-RMS	30 (9.0%)	1 (3.3%)	0	8 (26.7%)	2 (6.7%)
	nephroblastoma	13 (3.9%)	0	0	2 (15.4%)	2 (15.4%)
	carcinomas	13 (3.9%)	0	0	4 (30.8%)	2 (15.4%)
	Ewing sarcoma	6 (1.8%)	0	0	1 (16.7%)	0
	other malignancies ^†^	20 (6.0%)	2 (10.0%)	1 (5.0%)	2 (10.0%)	1 (5.0%)
	benign tumors ^‡^	7 (2.1%)	2 (25.0%)	0	2 (25.0%)	0

* TT—targeted therapy; NB—neuroblastoma, RMS—rhabdomyosarcoma; † germ-cell tumor (*n* = 3), hepatoblastoma (*n* = 2), melanoma/nevus (*n* = 4), neuroendocrine tumor (*n* = 1), gastrointestinal stromal tumor (*n* = 3), osteosarcoma (*n* = 3), pleuropulmonary blastoma (*n* = 3), malignant giant-cell tumor (*n* = 1). ‡ plexiform neurofibroma (*n* = 2), infantile myofibroma (*n* = 2), pneumocytoma (*n* = 1), lymphangioendothelioma (*n* = 1), ectomesenchymal chondromyxoid tumor (*n* = 1).

**Table 3:** Structure of TT recipients by demography, diagnosis, molecular markers and clinical parameters.

Parameter		Total	%
*n*		21	100
Sex	Male	14	66.7
	Female	7	33.3
Histological diagnosis	Neuroblastoma	13	62.0
	Myoepithelial carcinoma	2	9.5
	Ectomesenchymoma	1	4.75
	Spindle-cell undifferentiated sarcoma	1	4.75
	Melanoma	1	4.75
	Gastrointestinal stromal tumor	1	4.75
	Plexiform neurofibroma	1	4.75
	Infantile myofibromatosis	1	4.75
Line of therapy involving TT	1	7	33.35
	2	7	33.35
	3	5	23.8
	4	2	9.5
TT mode	Mono	8	38.1
	First-line	2	
	Second-line	6	
	Combination with chemotherapy	13	61.9
TT continuation, months	Mono	10.9 (0.8–43.5)	
	Combination	12.3 (0.3–61.5)	
Best response	Objective response CR + VGPR + PR *	10	47.6
	Mono	3/8	37.5
	Combination	7/13	53.8
	Stabilization	4	19.0
	Mono	3/8	37.5
	Combination	1/13	7.7
	Progression	7	33.3
Outcome	Sustained objective response	6	28.6
	Stabilization	3	14.2
	Progression	12	57.2
Progression incidence by actionability of predictive markers for TT	Tier IA	2	16.7
	Tier IB	8	66.7
	Tier IIC	1	8.3
	Tier IID	1	8.3

* CR, complete response; VGPR, very good partial response; PR, partial response.

## Data Availability

The additional data supporting the manuscript are available from the corresponding author upon request.

## Supplementary Materials

The following supporting information can be downloaded at https://www.mdpi.com/article/10.3390/ijms262311463/s1.

## Abbreviations

The following abbreviations are used in this manuscript:

NGS	Next-generation sequencing
TT	Targeted therapy
CT	Chemotherapy
NB	Neuroblastoma
FISH	Fluorescence In Situ Hybridization
MLPA	Multiplex Ligation-dependent Probe Amplification
CTCAE	Common Terminology Criteria for Adverse Events
RMS	Rhabdomyosarcoma
STS	Soft-tissue sarcoma
GIST	Gastrointestinal stromal tumor
MRT	Malignant rhabdoid tumor
CR	Complete response
VGPR	Very good partial response
PR	Partial response
HR	High-risk group
IR	Intermediate-risk group
LR	Low-risk group
MNA	*MYCN* amplification
