# Supplementary material for: Targeted DNA Sequencing for Tailored Therapies in Children with Extracranial Solid Tumors
Source: Int J Mol Sci. 2025 Nov 26;26(23):11463. doi: 10.3390/ijms262311463 (PMC12692115; doi:10.3390/ijms262311463)
Supplement: Supplementary file 1 [file ijms-26-11463-s001.zip › ijms-3963760-supplementary.pdf]

Gene panel for targeting DNA sequencing

Entire coding DNA sequence and canonical splice sites (minimum +10 bp of the intronic sequence)

ALK, APC, ATM, ATRX, BCOR, BRAF, BRCA1, BRCA2, CDKN2A, CDKN2B, CIC, CREBBP, CTNNB1, DICER1, DROSHA, EGFR, FBXW7, FGFR1, H3F3A, HISTH3B, HRAS, IDH1, IDH2, KDR, KIT, KRAS, MAP2K1, MAP2K2, MET, MLH1, MSH2, MSH6, MYOD1, NF1, NOTCH1, NRAS, PDGFRA, PDGFRB, PIK3CA, POLD1, POLE, PTCH1, PTEN, PTPN11, RB1, RET, ROS1, SMARCA4, SMARCB1, SMO, SUFU, TERT, TP53, TSC1, TSC2, WT1
